# Supplementary material for: Analysis of altered level of blood-based biomarkers in prognosis of COVID-19 patients
Source: PLoS One. 2023 Aug 4;18(8):e0287117. doi: 10.1371/journal.pone.0287117 (PMC10403103; doi:10.1371/journal.pone.0287117)
Supplement: Attachment — Submitted filename: PONE-D-22-06698_reviewer.pdf [file pone.0287117.s001.pdf]

PLOS ONE

Analysis of altered level of blood-based biomarkers in the prognosis of COVID-19 patients

--Manuscript Draft--

**Manuscript Number:**	PONE-D-22-06698
**Article Type:**	Research Article
**Full Title:**	Analysis of altered level of blood-based biomarkers in the prognosis of COVID-19 patients
**Short Title:**	Analysis of altered level of biomarkers in the prognosis of COVID-19 patients
**Corresponding Author:**	Ajaya Basnet, M.Sc.<br>Shi-Gan International College of Science and Technology<br>Kathmandu, Nepal NEPAL
**Keywords:**	Biomarkers, COVID-19, Nepal, Prognosis, SARS-CoV-2
**Abstract:**	Introduction: Immune and inflammatory responses developed by the patients with Coronavirus Disease 2019 (COVID-19) during rapid disease progression result in an altered level of biomarkers. Therefore, this study aimed to analyze levels of blood-based biomarkers that are significantly altered in patients with COVID–19.Methods: A cross-sectional study was conducted among COVID-19 diagnosed patients admitted to the tertiary care hospital. Several biomarkers – biochemical, hematological, inflammatory, cardiac, and coagulatory – were analyzed and subsequently tested for statistical significance at P<0.01 by using SPSS version 17.0.Results: A total of 1,780 samples were analyzed from 1,232 COVID-19 patients (median age 45 years [IQR 33-57]; 788 [63.96%] male). The COVID-19 patients had significantly (99% CI, p<0.001) elevated glucose, urea (p=0.001), alanine transaminase (ALT), aspartate aminotransaminase (AST), alkaline phosphatase (ALP), lactate dehydrogenase (LDH) levels, total white blood cell count (WBC), C-reactive protein (CRP), procalcitonin (PCT), interleukin-6 (IL-6), ferritin, D-Dimer, and creatinine phosphokinase-MB (CPK-MB, p=0.004) as compared to control group. However, the levels of total protein, albumin, and platelets were significantly lowered in COVID-19 patients as compared to control group. The elevated levels of glucose, urea, direct bilirubin, WBC, CRP, prothrombin time (PT), D-Dimer, and LDH were significantly associated with in-hospital mortality among COVID-19 patients.Conclusions: Assessing and monitoring the elevated levels of glucose, urea, ALT, AST, ALP, ferritin, DB, WBC, CRP, PCT, LDH, D-Dimer, PT, and CPK-MB and the lowered levels of total protein, albumin, and platelet could provide a basis for evaluation of improved prognosis and effective treatment in patients with COVID-19.
**Order of Authors:**	Mahendra Raj Shrestha, MD
	Ajaya Basnet, MSc
	Basanta Tamang, MSc
	Sudip Khadka, PhD
	Rajendra Maharjan, MD
	Rupak Maharjan, MD
	Arun Bahadur Chand, MSc
	Suresh Thapa, BMLT
	Shiba Kumar Rai, MSc
**Additional Information:**	
**Question**	**Response**
**Financial Disclosure**<br><br>Enter a financial disclosure statement that	The author(s) received no specific funding for this work.

describes the sources of funding for the work included in this submission. Review the submission guidelines for detailed requirements. View published research articles from *PLOS ONE* for specific examples.<br><br>This statement is required for submission and **will appear in the published article** if the submission is accepted. Please make sure it is accurate.<br><br>**Unfunded studies**<br>Enter: *The author(s) received no specific funding for this work.*<br><br>**Funded studies**<br>Enter a statement with the following details:<br>• Initials of the authors who received each award<br>• Grant numbers awarded to each author<br>• The full name of each funder<br>• URL of each funder website<br>• Did the sponsors or funders play any role in the study design, data collection and analysis, decision to publish, or preparation of the manuscript?<br>• **NO** - Include this sentence at the end of your statement: *The funders had no role in study design, data collection and analysis, decision to publish, or preparation of the manuscript.*<br>• **YES** - Specify the role(s) played.<br><br>* typeset	
**Competing Interests**<br><br>Use the instructions below to enter a competing interest statement for this submission. On behalf of all authors, disclose any competing interests that could be perceived to bias this work—acknowledging all financial support and any other relevant financial or non-financial competing interests.<br><br>This statement is required for submission and **will appear in the published article** if the submission is accepted. Please make sure it is accurate and that any funding	The authors have declared that no competing interests exist.

sources listed in your Funding Information later in the submission form are also declared in your Financial Disclosure statement.<br><br>View published research articles from *PLOS ONE* for specific examples.<br><br>**NO authors have competing interests**<br><br>Enter: *The authors have declared that no competing interests exist.*<br><br>**Authors with competing interests**<br><br>Enter competing interest details beginning with this statement:<br><br>*I have read the journal's policy and the authors of this manuscript have the following competing interests: [insert competing interests here]*<br><br>* typeset	
**Ethics Statement**<br><br>Enter an ethics statement for this submission. This statement is required if the study involved:<br><br>• Human participants<br>• Human specimens or tissue<br>• Vertebrate animals or cephalopods<br>• Vertebrate embryos or tissues<br>• Field research<br><br>Write "N/A" if the submission does not require an ethics statement.<br><br>General guidance is provided below. Consult the submission guidelines for detailed instructions. **Make sure that all information entered here is included in the Methods section of the manuscript.**	This study was ethically approved by Shi-Gan health Foundation, Shankhamarg, Chakrapth, Kathmandu, Nepal.<br>Verbal consent was obtained from all patients with COVID-19, as the patients were contagious for the virus transmissible, when used physical method.

**Format for specific study types**<br><br>**Human Subject Research (involving human participants and/or tissue)**<br>• Give the name of the institutional review board or ethics committee that approved the study<br>• Include the approval number and/or a statement indicating approval of this research<br>• Indicate the form of consent obtained (written/oral) or the reason that consent was not obtained (e.g. the data were analyzed anonymously)<br><br>**Animal Research (involving vertebrate animals, embryos or tissues)**<br>• Provide the name of the Institutional Animal Care and Use Committee (IACUC) or other relevant ethics board that reviewed the study protocol, and indicate whether they approved this research or granted a formal waiver of ethical approval<br>• Include an approval number if one was obtained<br>• If the study involved *non-human primates*, add *additional details* about animal welfare and steps taken to ameliorate suffering<br>• If anesthesia, euthanasia, or any kind of animal sacrifice is part of the study, include briefly which substances and/or methods were applied<br><br>**Field Research**<br><br>Include the following details if this study involves the collection of plant, animal, or other materials from a natural setting:<br>• Field permit number<br>• Name of the institution or relevant body that granted permission	
**Data Availability**<br><br>Authors are required to make all data underlying the findings described fully available, without restriction, and from the time of publication. PLOS allows rare exceptions to address legal and ethical concerns. See the PLOS Data Policy and FAQ for detailed information.	Yes - all data are fully available without restriction

A Data Availability Statement describing where the data can be found is required at submission. Your answers to this question constitute the Data Availability Statement and **will be published in the article**, if accepted.<br><br>**Important:** Stating 'data available on request from the author' is not sufficient. If your data are only available upon request, select 'No' for the first question and explain your exceptional situation in the text box.<br><br>Do the authors confirm that all data underlying the findings described in their manuscript are fully available without restriction?	
**Describe where the data may be found in full sentences. If you are copying our sample text, replace any instances of XXX with the appropriate details.**<br><br>• If the data are **held or will be held in a public repository**, include URLs, accession numbers or DOIs. If this information will only be available after acceptance, indicate this by ticking the box below. For example: *All XXX files are available from the XXX database (accession number(s) XXX, XXX.)*.<br>• If the data are all contained **within the manuscript and/or Supporting Information files**, enter the following: *All relevant data are within the manuscript and its Supporting Information files.*<br>• If neither of these applies but you are able to provide **details of access elsewhere**, with or without limitations, please do so. For example:<br><br>*Data cannot be shared publicly because of [XXX]. Data are available from the XXX Institutional Data Access / Ethics Committee (contact via XXX) for researchers who meet the criteria for access to confidential data.*<br><br>*The data underlying the results presented in the study are available from (include the name of the third party*	The data underlying the results presented in the study are available from the corresponding author Mr. Ajaya Basnet, upon the request.

and contact information or URL).<br>• This text is appropriate if the data are owned by a third party and authors do not have permission to share the data.<br><br>* typeset	
Additional data availability information:	

Cover Letter

Click here to access/download;Manuscript;Biomarkers Manuscript.docx

Analysis of altered level of blood-based biomarkers in the prognosis of COVID-19 patients

Mahendra Raj Shrestha[1], Ajaya Basnet[2]*, Basanta Tamang[1], Sudip Khadka[3], Rajendra Maharjan[1], Rupak Maharjan[1], Arun Bahadur Chand [4], Suresh Thapa[1], Shiba Kumar Rai[5]

[1] Department of Clinical Laboratory, Nepal Armed Police Force Hospital, Kathmandu, Bagmati, Nepal

[2] Department of Medical Microbiology, Shi-Gan International College of Science and Technology, Tribhuvan University, Kathmandu, Bagmati, Nepal

[3] Department of Immunology, Mayoclinic, SW Rochester, United States

[4] Department of Clinical Laboratory, KIST Medical College and Teaching Hospital, Lalitpur, Bagmati, Nepal

[5] Research Department, Nepal Medical College Teaching Hospital, Kathmandu, Bagmati, Nepal

* Corresponding author

E-mail: xlcprk@gmail.com (AB)

Abstract

Introduction: Immune and inflammatory responses developed by the patients with Coronavirus Disease 2019 (COVID-19) during rapid disease progression result in an altered level of biomarkers. Therefore, this study aimed to analyze levels of blood-based biomarkers that are significantly altered in patients with COVID–19.

Methods: A cross-sectional study was conducted among COVID-19 diagnosed patients admitted to the tertiary care hospital. Several biomarkers – biochemical, hematological, inflammatory, cardiac, and coagulatory – were analyzed and subsequently tested for statistical significance at P<0.01 by using SPSS version 17.0.

Results: A total of 1,780 samples were analyzed from 1,232 COVID-19 patients (median age 45 years [IQR 33-57]; 788 [63.96%] male). The COVID-19 patients had significantly (99% CI, p<0.001) elevated glucose, urea (p=0.001), alanine transaminase (ALT), aspartate aminotransaminase (AST), alkaline phosphatase (ALP), lactate dehydrogenase (LDH) levels, total white blood cell count (WBC), C-reactive protein (CRP), procalcitonin (PCT), interleukin-6 (IL-6), ferritin, D-Dimer, and creatinine phosphokinase-MB (CPK-MB, p=0.004) as compared to control group. However, the levels of total protein, albumin, and platelets were significantly lowered in COVID-19 patients as compared to control group. The elevated levels of glucose, urea, direct bilirubin, WBC, CRP, prothrombin time (PT), D-Dimer, and LDH were significantly associated with in-hospital mortality among COVID-19 patients.

Conclusions: Assessing and monitoring the elevated levels of glucose, urea, ALT, AST, ALP, ferritin, DB, WBC, CRP, PCT, LDH, D-Dimer, PT, and CPK-MB and the lowered levels of total protein, albumin, and platelet could provide a basis for evaluation of improved prognosis and effective treatment in patients with COVID-19.

Keywords: Biomarkers, COVID-19, Nepal, Prognosis, SARS-CoV-2.

Introduction

Ever since the first case of COVID-19 caused pneumonia due to Severe Acute Respiratory Syndrome Coronavirus-2 (SARS-CoV-2), an enveloped RNA beta *Coronavirus* was reported in Wuhan, Hubei Province, China in December 2019 [1,2], the viral infection spread rapidly around the world and subsequently reached pandemic level [3], as declared by World Health Organization (WHO) on March 11, 2020 [4]. As of August 21, 2021, there are over 211 million confirmed cases of COVID-19 with more than 4 million deaths, and still surging across 217 countries worldwide [5]. In Nepal, 7,44,307 people have been diagnosed with COVID-19 with a cumulative death of 10,455 people until August 21, 2021 [6].

The clinical course of COVID-19 is often unpredictable and is characterized by oligosymptomatic forms of the disease. It can present itself as an asymptomatic or mild case (30-60%) to moderate [7] or severe/critical case, which is often characterized by pneumonia (6%) [8]. Severe case may evolve into acute respiratory distress syndrome (ARDS) and systemic illness involving multiple systems [9], that potentially leads to death [1,3]. Elderly (>65 years), male sex, and individuals with comorbidities- such as diabetes, hypertension, cardiovascular diseases, and chronic obstructive pulmonary disease [3,10] are often predisposed to the severity of COVID-19 [7,11].

Since the beginning, COVID-19 has exerted persistent pressure on health care systems to categorize patients into risk groups following diagnosis [12]. Though the diagnosis of COVID-19 with a real-time polymerase chain reaction (RT-PCR) [12] can be performed for an extremely sensitive and specific result; however, considering the facts of false-negative result and the increased expenses, complexity, and time; the reliability of RT-PCR has been limited [13]. Moreover, to this date, there is no standard therapy modality for COVID-19 patients [14]. Due to uncertain nature of disease progression events and a potential for a

patient to present severe symptoms in a short period of time it is important to explore early predictors of disease progression to provide timely intervention [14]. Therefore, identification of an alternative methodology to predict outcomes, preferably a set of biomarkers, which could aid in risk stratification of COVID-19 patients, is highly desirable [15]. These predictors can potentially identify patients likely to present severe disease earlier, classify the disease severity, frame hospital/ICU admission criteria, and more importantly, frame the criteria for patient discharge [2].

The clinical course of COVID-19 can be classified as (a) early infection marked by infection of ciliated broncho-epithelial cells mediated by the interaction of SARS-CoV-2 spike glycoprotein (S) with the angiotensin-converting enzyme 2 (ACE2) (b) pulmonary phase characterized by viral pneumonia associated with localized inflammation within the lung, and (c) hyper inflammation phase indicated by systemic inflammation or cytokine storm [3] that includes increased concentrations of different types of cytokines (IFN-γ, TNF-α, IL-2, IL-6, IL-7, IL-10 and others) [3,11]. Increased inflammation in patients may result in systemic vasculitic processes and defects in the coagulation processes [12]. The deranged levels of electrolyte (sodium, potassium, and calcium), liver enzymes, inflammatory mediators (interleukins, C-reactive proteins), coagulation markers (fibrinogen and prothrombin time), and cardiac biomarkers (troponin, CPK-MB) among patients with COVID-19 could be correlated with disease severity and/or with high mortality rates [16].

While there have been copious studies concerning disease severity, vaccines, and secondary bacterial infections associated with COVID-19, to date, there has been a limited studies, involving relatively small patient cohorts, concerning the role of clinical biomarkers for the prognosis of SARS-CoV-2 infected patients. Therefore, this study aims to provide practical information to the clinicians on the role of biochemical, hematological, inflammatory,

cardiac, and coagulatory biomarkers, focusing on those potentially predictive of organ damage, in COVID-19 patients admitted in a tertiary care hospital.

Methodology

Study design and population

A cross-sectional study was conducted on hospitalized COVID-19 patients at COVID-19 dedicated Nepal Armed Police Force Hospital (NAPFH), Kathmandu, Nepal. COVID-19 patients, confirmed positive by reverse transcriptase-polymerase chain reaction (RT-PCR) of nasopharyngeal and oropharyngeal swab samples, of any age and gender admitted to the hospital from April 14, 2021, to September 14, 2021, were included in this study. All COVID-19 non-confirmed cases and patients on corticosteroid therapy were excluded from this study.

Data collection

After the approval of Institutional Review Committee from Shi-Gan Health Foundation, the relevant demographic data, medical history, laboratory findings, etc. of COVID-19 patients were initially recorded in the patient information sheet, and later in the Microsoft Excel. Any missing or ambiguous records were gathered and clarified through communication with involved healthcare workers or patients and their families.

Laboratory analysis

Our study examined several biomarkers: biochemical parameters (glucose, urea, TB, DB, ALT, AST, ALP, total protein, albumin, and LDH); hematological parameters (hemoglobin, WBC, and platelets); inflammatory biomarkers (CRP, PCT, IL-6, and ferritin); coagulatory biomarkers (PT and D-dimer); and cardiac biomarkers (troponin, CPK-MB). The D dimer, CPK-MB, ferritin, CRP, PCT, and IL-6 were analyzed by (Fine Care Fluorescence Analyzer, China). All of the biochemistry biomarkers were analyzed by Diatron Fully Automated

Biochemistry Analyzer (Model: Pictus 500), except for sodium and potassium, which were measured by Medica EasyLyte Analyzer (United States). The hematological biomarkers were analyzed by Beckman Coulter (Model: DxH 500).

Statistical methods

Continuous and categorical variables were presented as mean ± standard deviation. We used two independent samples' *t*-test of SPSS version 17.0 software (SPSS Inc., Chicago, IL, USA) to compare differences between COVID-19 patients and the control. A *P*-value less than 0.01 was considered statistically significant.

Results:

Demographics

Among a total of 1,732 patients included in the analysis, 1,232 (78.07%) were COVID-19 patients and 500 (21.93%) were the control. There were 788 (63.96%) male COVID-19 patients. The COVID-19 patients had a mean age of 47.25 years ± 25.65 (Range 5-94 years). The control group was also predominated with males (*n* = 314, 62.80%) with mean age of 41.26 years ± 22.44 (IQR 24–58.75). Among the COVID-19 patients, age group of ≥60 years (*n* = 269, 21.83%) were predominant, followed by patients of age group 40-50 years (*n* = 268, 21.75%), 50-60 years (*n* = 237, 19.24%), 30-40 years (*n* = 229, 18.59%), 20-30 years (*n* = 211, 17.13%), 10-20 years (*n* = 16, 1.30%), and <10 years (*n* = 2, 0.16%). While the mean age of COVID-19 patients was statistically significant (p<0.001), the gender (p=0.650) was not statistically significant between the two groups. Concerning the disease outcome, 58 (4.71%) died while 1,174 (95.29%) recovered (Table 1).

Table 1: Patients distribution based on age group, gender, and in-hospital death

	**COVID-19** (*n* = 1,232)	**Control** (*n* = 500)	**P-value**
**Age (years)**	47.25 ± 25.65	41.26 ± 22.44 years	<0.001
< 10	2	62	
10-20	16	28	
20-30	211	69	
30-40	229	67	
40-50	268	74	
50-60	237	78	
≥ 60	269	122	
**Gender**			
Male	788	314	0.650
Female	444	186	

Deceased			
Yes	58	-	<0.001
No	1,174	-	

Biomarkers

There were significant differences between the groups in the mean ± standard deviation levels for most of the biomarkers examined.

(a) Biochemistry

The biochemical abnormalities found in COVID-19 patients are shown in Table 2. The COVID-19 patients had significantly elevated glucose (169.83 mg/dL ± 86.29) (p=0.001), urea (60.16 mg/dL ± 42.29) (p=0.001), ALT (69.13 U/L ± 92.35) (p=0.001), AST (57.66 U/L ± 83.74) (p=0.003), ALP (114.84 U/L± 76.40) (p=0.001), and LDH (951.34 µg/L ± 505.39) (p=0.001) levels as compared to the glucose (103.63 mg/dL ± 33.26), urea (36.66 mg/dL ± 36.59), ALT (47.14 U/L ± 36.85), AST (46.14 U/L ± 43.58), ALP (92.55 U/L ± 41.76), and LDH (629.66 µg/L ± 892.16) levels observed in the control. A noteworthy increase in serum sodium (140.71 mEq/L ± 39.37) (p=0.167), creatinine (1.52 mg/dL ± 6.52) (p=0.320) and total bilirubin (1.05 mg/dL ± 0.99) (p=0.696) levels was also observe in the COVID-19 patients. In contrast, a decreased level in serum potassium (4.07 mmol/L ± 5.46) (p=0.012), direct bilirubin (0.34 mg/dL ± 0.47) (p=0.015), total protein (6.01 g/dL ± 2.39) (p=0.001), and albumin (3.12 g/dL ± 0.54) (p=0.001) was observed in the patients with COVID-19 as compared to serum creatinine (1.34 mg/dL ± 1.95), potassium (5.52 mmol/L ± 19.94), direct bilirubin (0.44 mg/dL ± 1.21), total protein (7.20 g/dL ± 0.91), and albumin (3.62 g/dL ± 0.87) levels observed in the control group. Lastly, an indicator of tissue damage, namely LDH (951.34 U/L ± 505.39) (p=0.001), was found to be significantly increased in COVID-19 patients as compared to that of control group (629.66 ± 892.16) (Table 2).

Table 2: Analysis of biomarkers for samples received from COVID-19 and control

	**COVID-19** (*n* = 1780)	**Control** (*n* = 500)	**P-value**
**Biochemistry**			
Glucose (mg/dL) (*n* = 1498)	169.83 ± 86.29	103.63 ± 33.26	0.001
Urea (mg/dL) (*n* = 1652)	60.16 ± 42.29	36.66 ± 36.59	0.001
Creatinine (mg/dL) (*n* = 1534)	1.52 ± 6.52	1.34 ± 1.95	0.320
Sodium (mEq/L) (*n* = 1634)	140.71 ± 39.37	139.15 ± 12.56	0.167
Potassium (mmol/L) (*n* = 1483)	4.07 ± 5.46	5.52 ± 19.94	0.012
Total Bilirubin (mg/dL) (*n* = 1399)	1.05 ± 0.99	1.03 ± 2.02	0.696
Direct Bilirubin (mg/dL) (*n* = 1379)	0.34 ± 0.47	0.44 ± 1.21	0.015
Alanine transaminase (U/L) (*n* = 1498)	69.13 ± 92.35	47.14 ± 36.85	0.001
Aspartate aminotransaminase (U/L) (*n* = 1498)	57.66 ± 83.74	46.14 ± 43.58	0.003
Alkaline phosphatase (U/L) (*n* = 1452)	114.84 ± 76.40	92.55 ± 41.76	0.001
Total protein (g/dL) (*n* = 574)	6.01 ± 2.39	7.20 ± 0.91	0.001
Albumin (g/dL) (*n* = 618)	3.12 ± 0.54	3.62 ± 0.87	0.001
Lactate dehydrogenase (U/L) (*n* = 279)	951.34 ± 505.39	629.66 ± 892.16	0.001
**Hematology**			
Hemoglobin (g/dL) (*n* = 1259)	13.45 ± 4.52	13.99 ± 9.92	0.121
WBC (billion cells/L) (*n* = 1334)	11.82 ± 6.81	8.12 ± 3.52	0.001
Platelet (billion/L) (*n* = 1333)	226.46 ± 100.91	251.17 ± 91.30	0.001

**Inflammatory**			
C-Reactive Protein (mg/L) (*n* = 1127)	71.17 ± 65.15	16.73 ± 38.33	0.001
Procalcitonin (ng/ml) (*n* = 508)	3.32 ± 11.12	1.06 ± 2.95	0.001
Interleukin-6 (pg/ml) (*n* = 15)	618.61 ± 987.29	1.6 [NR]	0.001
Ferritin (µg/L) (*n* = 62)	445.36 ± 281.88	91.31 ± 68.63	0.001
**Coagulatory**			
Prothrombin Time (sec) (*n* = 592)	16.36 ± 53.47	14.71 ± 4.46	0.492
D-Dimer (ng/mL) (*n* = 1083)	2.09 ± 2.76	0.83 ± 3.89	0.001
**Cardiac**			
Troponin (ng/L) (*n* = 50)	-	-	-
Creatinine phosphokinase-MB (U/L) (*n* = 58)	29.87 ± 39.49	14.40 ± 15.52	0.001
NR: Normal Range			

(b) Hematology

Among the hematological parameters, though there was a significant elevation in total WBC count (11.82 billion cells/L ± 6.81) (p=0.001) in COVID-19 patients, there was also a significant decrease in platelet count (226.46 billion/L ± 100.91 (p=0.001) as compared to the WBC count (8.12 billion cells/L ± 3.52) and platelet count (251.17 billion/L ± 91.30) in the control group. Additionally, we also observed a modest decrease in total hemoglobin levels (13.45 g/dL ± 4.52) (p=0.121) in COVID-19 patients as compared to that of control group (13.99 g/dL ± 9.92) (Table 2).

(c) Inflammatory

We also analyzed various biomarkers of inflammatory response. The majority of COVID-19 patients showed significantly higher levels of CRP (71.17 mg/L ± 65.15) (p=0.001), PCT (3.32 ng/ml ± 11.12) (p=0.001), and ferritin (445.36 µg/L ± 281.88) (p=0.001) as compared to that of control cohort; CRP (16.73 mg/L ± 38.33), PCT (1.06 ng/ml ± 2.95), and ferritin (91.31 µg/L ± 68.63). Moreover, significantly elevated IL-6 levels (618.61 pg/ml ± 987.29) in COVID-19 patients than basal level (1.6 pg/ml) potentiate IL-6 levels as a strong indicator of the severity of COVID-19 infection (Table 2).

(d) Coagulatory

Coagulatory biomarker, namely, D-Dimer (2.09 ng/mL ± 2.76) (p=0.001) was significantly higher in COVID-19 patients than that of control group (0.83 ng/mL ± 3.89). Notably, our analysis of prothrombin time (16.36 sec ± 53.47) (p=0.492) in COVID-19 patients showed an insignificant increment as compared to the prothrombin time (14.71 sec ± 4.46) of the control group (Table 2).

(e) Cardiac

The COVID-19 patients showed significantly elevated levels of CPK-MB (29.87 U/L ± 39.49) (p=0.001) as compared to the CPK-MB (14.40 U/L ± 15.52) of the control. Moreover, the qualitative analysis of troponin test in both the group gave the negative result (Table 2).

The COVID-19 patients (n = 58) with elevated levels of glucose (p=0.007), urea (p=0.009), direct bilirubin (DB; p=0.049), WBC (p=0.001), CRP (p=0.004), PT (p=0.001), D-Dimer (p=0.001), and LDH (p=0.030) died due to COVID-19 and its associated complications.

Discussion

The ongoing COVID-19 pandemic has placed a massive burden on the global healthcare system [17]. COVID-19 positive patients, can present mild to moderate symptoms and within a short period of time can manifest a severe form of disease. A lack of prediction capability as to which cases are likely to progress onto severe forms has been a huge challenge in proper management of positive cases. Hence, it is imperative that a methodology to identify patients with a potential to present severe symptoms utilizing laboratory indices of disease progression is desirable. Such a methodology can effectively monitor the prognosis of high-risk patients [14,15]. Blood biomarkers can provide valuable prognostic information for patients diagnosed with COVID-19 in a timely manner [2]. In this study, we analyzed the variations in the levels of several biomarkers of biochemical, hematological, inflammatory, cardiac, and coagulatory processes and identified their association with COVID-19 patients.

WHO [18] and the Center for Disease Control and Prevention (CDC) [19] mention a higher risk for older people to contract SARS-CoV-2 infection. Elderly patients often are in need of hospitalization with intensive care facilities and/or a ventilator. This study also found that COVID-19 patients with age of ≥60 years (21.83%) were at a higher risk of COVID-19 infection, which was similar to the findings of several other research [7,14,20]. Some of the reasons put forward for this is due to gradual weakening of the immune system, age-related modification in the respiratory system, and the presence of co-morbidities such as hypertension, diabetes, chronic obstructive pulmonary diseases and others [21]. The mean age of COVID-19 patients (47.25 years) in our study was comparable with the findings from other studies [22-27]. However, several studies have discussed the different mean age groups in COVID-19 patients, ranging from 32.5 to 76 years [28-31]. In this study,nearly two-third of infection was seen in males and was consistent with the findings of Zhou et al. [32],

Colaneri et al. [33], Goyal et al. [34] and several other studies [23,27].This might be attributed to the adaptive immune system of females, who have higher numbers of CD4+ T cells, more robust CD8+ T cell cytotoxic activity, and increased B cell production of immunoglobulin as compared to males [35]. However, these phenomena are poorly understood and further studies are needed to understand the predilection of COVID-19 infection in males.

We observed altered liver function biomarkers in COVID-19 patients. Alteration in levels of liver dysfunction biomarkers has been associated with increased severity and a worse prognosis of COVID-19 by several independent studies [3,17]. As reported by Fan et al. [36], 50.7% of patients (n=148) had an aberrant liver function upon admission, primarily with an increase in Gamma-glutamyl Transferase (GGT), ALP, AST, and ALT levels. This finding is consistent with our study, which shows an increase in levels of total bilirubin ($P>0.01$), AST ($P<0.01$), ALP ($P<0.01$), ALT ($P<0.01$), and lowered levels of direct bilirubin ($P>0.01$) among COVID-19 patients as compared to the control group. Similar to our findings, a study performed by Cai et al. [37] in a cohort of 417 COVID-19 patients showed that 76.3% of patients had abnormal liver tests, defined by elevated levels (more than 3 times the upper limit of normal) of ALT, AST, total bilirubin and GGT. The same study also found that 21.5% of patients with abnormal liver tests had developed liver injury and had higher odds of developing severe pneumonia [37]. COVID-19-related liver abnormalities could be attributed either to the direct binding of SARS-CoV-2 with ACE2 receptor present in bile duct epithelial cells, namely cholangiocytes [3], or to other causes, such as administration of hepatotoxic drugs, hypoxia associated hepatocellular necrosis, and systemic inflammatory response [12]. A retrospective study conducted by Lei et al. [38] involving a data set of 5771 COVID-19 patients reported that AST is strongly associated with mortality risk. Similarly, Chen et al. [39] observed markedly higher concentrations of both ALT and AST in non-

survivors compared to recovered patients. One of the explanations presented for elevated transaminases was attributed to potential hepatic congestion due to right heart dysfunction in the setting of high pulmonary pressures in intubated ARDS patients [17].

Furthermore, our study showed a significant association of lowered levels of albumin (3.12 g/dL± 0.54) (P<0.01) and total protein (6.01 g/dL± 2.39) (P<0.01) in COVID-19 patients as compared to the control group which showed the levels of3.62 g/dL± 0.87 and 7.20g/dL ± 0.91 respectively. Considerably higher level of albumin observed in the improvement/ stabilization group than that in the disease progression group (41.27 g/dL ± 4.55 vs. 36.62 g/dL ± 6.60) was also observed by Liu et al. [40]. Similarly, a meta-analysis of 11 studies showed that the mean serum albumin on admission was 3.50 g/dl and 4.05 g/dl in severe and non-severe COVID-19, respectively [41], which could be compared with the findings from our study.

According to an Italian Report, about 25–30% of COVID-19 patients develops acute kidney injury (AKI) [42] and become more susceptible to mortality [3]. This study also analyzed key indicators of kidney function. This study showed an increased level of glucose, urea and sodium and decreased levels of creatinine and potassium among COVID-19 patients as compared to the control group. However, several studies [3,15] have also reported that elevated serum creatinine levels increase the chance of AKI and other poor outcomes, including death of patients. Zhou et al. [32] reported an increased level of blood urea nitrogen in 2.8% of the patients, indicating “kidney dysfunction” in a few cases. It has been thought that the hematogenous spread and direct interaction of virus with ACE2 receptor present in the cellular surface of kidney tubular cells resulting in endothelial dysfunction, microcirculatory derangement, and tubular injury [3].

Our result demonstrated a significantly higher LDH value in COVID-19 patients as compared to the control group. The finding was in consensus with a pooled analysis from the study of

Henry et al. [43], which revealed elevated LDH values associated with a >16-fold increase in odds of mortality and a 6-fold increased odds of severe disease. Several other studies have also shown a significant correlation between an increase in LDH level with an increase in disease severity [44,45]. Elevated LDH levels might reduce the effectiveness of lactic acid and lead to tissue injury, especially of the cardiac, pulmonary, and hepatic origin [2,15,14], via the action of metalloproteinases and enhanced macrophage-mediated angiogenesis [15].

Leucocytosis is another alteration detectable at the complete blood count of COVID-19 patients [3]. This study showed an increased leukocytes count in COVID-19 patients as compared to control. Several other studies concluded that patients with severe and fatal cases tend to have increased leukocyte counts compared to patients with mild cases [46,47]. In this study, hemoglobin and platelets were substantially reduced in COVID-19 patients. The decreased number of platelets with increased risk of severe disease and mortality for COVID-19 patients has been reported in several studies [48,49]. The reduction of platelet count in the COVID-19 patients could be attributed to the activation, aggregation, and formation of microthrombi, which leads to decreased platelet production and increased consumption [3,12]. However, Yin et al. [50] and Qu et al. [51] reported that patients with COVID-19 had higher platelet count than those in non-COVID-19 patients, and concluded that severe thrombocytopenia and bleeding are uncommon in patients with COVID-19. Hence, a more thorough study is warranted to understand the nature of relationship between platelet count and COVID severity.

This study showed an absolute negative result for troponin and elevated level for CPK-MB ($P<0.01$) in COVID-19 patients. However, several studies [32,52] have associated the increased level of troponin with severe illness and predictive of mortality as compared to those without severe illness (25.6 ng/L vs 6.8–44.5 ng/L) [53]. An insignificant association of troponin among COVID-19 patients from our study could be due to poor sample size (*n*= 50),

and qualitative analysis of the test result, and unlikely due to the quantitative measurements as performed in several researches. Other studies have reported that elevated CK levels are associated with a nearly fourfold increase in poor outcomes in COVID-19 patients, which is consistent with the findings from our study [54,55]. Cardiac injuries in COVID-19 patients could be accredited to the direct viral infection of ACE2 receptor present in myocardial cells, cardiac stress due to respiratory failure and hypoxemia, and systemic inflammatory response [3]. Moreover, myositis could also have occurred either due to viral-mediated or immune-mediated damage of myocytes, including deposition of virus–antibody complexes in muscles and/or circulating viral toxins in the blood [55].

In this study, inflammatory biomarkers such as CRP, PCT, and IL-6 in COVID-19 patients were found significantly elevated as compared to control group. Various studies showed that CRP increases significantly in severe COVID–19 patients at the early stage of the disease development and concluded that elevated CRP levels could be used as the most sensitive biomarker in predicting the disease progression [3,56-59]. Moreover, it has been shown that a cut-off value of >10 mg/L for CRP and >0.5 ng/ml for PCT helpful to predict progression to severe disease [2]. Those studies showed that while the circulating PCT value remains within the normal range (<0.1 ng/mL) in non-complicated COVID-19 patients, the elevated levels reflect systemic bacterial coinfection and progression towards more severe complications [3,15,59] with a 5-fold higher risk of severe infection [49]. Our study documented serum ferritin level to be significantly increased in COVID-19 patients as compared to control. Ponti et al. [12] reported significantly increased ferritin levels in non-survivors as compared to survivors and concluded that a patient with an elevated ferritin level has a high probability to experience serious inflammatory complications. Since, the ensuing innate immune pro-inflammatory response and the adaptive immune dysregulated response of the host results in an imbalance favoring naive T cell activity against regulatory T cells, this leads to

hyperinflammation through a massive, coordinated release of cytokines [2]. Additionally, cytokine storm could also be triggered via several pathways, including the NF- kB, JAK/STAT, and the macrophage activation pathway leading to the release of IL-6 and TNF-**α** [2].

An increased inflammatory response and hypoxia due to severe pneumonia in COVID-19 patients eventually lead to blood coagulation function disorders, including disseminated intravascular coagulation (DIC) [15]. In this study, we found significantly elevated D-dimer value and prothrombin times are observed in COVID-19 patients. Zhou et al. [32] reported that D-dimer levels greater than 1.0 μg/mL on admission increased in-hospital mortality of COVID-19 patients; Zhang et al. [60] reported the value greater than 2.0 μg/mL on admission, which was similar to the value (2.09 ± 2.76) from present study. Several other studies have discussed that elevated levels of D-dimer were significantly associated with an increased risk of poor outcomes by upto threefold [14,61-63]. Moreover, Wang et al. [64] showed that 58% of patients with COVID-19 had prolonged PT. Various studies have positively correlated higher PT in COVID-19 patients at the time of admission [9,14,63,65].Lastly, the deceased patients in our study had elevated levels of glucose, urea, DB, WBC, CRP, PT, D-Dimer and LDH (Table 2), which reflected the pathobiological axes of tissue injury, coagulation, and inflammation and hence we summarized that these biomarkers could be used to predict patient prognosis better at an early stage.

This study suffers from several limitations. This was a single-center cross-sectional study comprising the Nepalese population who visited the study site seeking medical care. Our findings were based on a limited number of observational studies; therefore, further well-designed studies with larger sample sizes are necessary to understand the effectiveness of biomarkers for prognosis of COVID-19 patients.

Conclusion

The progression of SARS-CoV-2 infection is characterized by alteration of several blood biomarkers, which may contribute to a person's illness, disease severity, and likelihood of death. Our analysis suggests that biochemical (glucose, urea, ALT, AST, ALP, total protein, albumin, TB, LDH), hematological (leucocytes, thrombocytes), inflammatory (CRP, PCT, IL-6, ferritin), coagulatory (PT, D-dimer), and cardiac (CPK-MB) represent the most significant parameters for the prognosis of patients with COVID-19. Detection and monitoring of a panel of such disease-specific indices will allow clinicians to properly manage high-risk COVID-19 patients, potentially improve prognosis, and reduce mortality rate.

Acknowledgments

We would like to take the privilege of acknowledging the dedicated laboratory personnel of NAPFH for their cooperation and patience in carrying out this endeavor.

Reference

1. Cannata F, Bombace S, Stefanini GG. Cardiac biomarkers in patients with COVID-19: pragmatic tools in hard times. Revista Española de Cardiología (English Edition). 2021 Jan 21.

2. Samprathi M, Jayashree M. Biomarkers in COVID-19: An up-to-date review. Frontiers in Pediatrics. 2020;8.

3. Ciaccio M, Agnello L. Biochemical biomarkers alterations in Coronavirus Disease 2019 (COVID-19). Diagnosis. 2020 Dec 1;7(4):365-72.

4. World Health Organization. Coronavirus disease (COVID-19) outbreak. 2020. [Cited 30 September 2021]. Available from: https:// www.who.int/emergencies/diseases/novel-coronavirus-2019

5. Worldometer. [Cited 30 September 2021]. Available from: https://www.worldometers.info/coronavirus/?fbclid=IwAR35ZFiRZJ8tyBCwazX2N-k7yJjZOLDQiZSA_MsJAfdK74s8f2a_Dgx4iVk

6. Ministry of Health and Population. Nepal. [Cited 30 September 2021]. Available from: https://covid19.mohp.gov.np/

7. Li X, To KK. Biomarkers for severe COVID-19. EBioMedicine. 2021 Jun 1;68.

8. Santos Filho LR, RB AT. Covid-19 and endocrinology–intersections: a review of current knowledge on physiopathological associations. Endocrinol Metab Int J. 2020;8(4):90-5.

9. Terpos E, Ntanasis-Stathopoulos I, Elalamy I, Kastritis E, Sergentanis TN, Politou M, Psaltopoulou T, Gerotziafas G, Dimopoulos MA. Hematological findings and complications of COVID-19. American journal of hematology. 2020 Jul;95(7):834-47.

10. Tian J, Yuan X, Xiao J, Zhong Q, Yang C, Liu B, Cai Y, Lu Z, Wang J, Wang Y, Liu S. Clinical characteristics and risk factors associated with COVID-19 disease severity in patients with cancer in Wuhan, China: a multicentre, retrospective, cohort study. The Lancet Oncology. 2020 Jul 1;21(7):893-903.

11. Velavan TP, Meyer CG. Mild versus severe COVID-19: laboratory markers. International Journal of Infectious Diseases. 2020 Jun 1;95:304-7.

12. Ponti G, Maccaferri M, Ruini C, Tomasi A, Ozben T. Biomarkers associated with COVID-19 disease progression. Critical reviews in clinical laboratory sciences. 2020 Aug 17;57(6):389-99.

13. Afzal A. Molecular diagnostic technologies for COVID-19: Limitations and challenges. Journal of advanced research. 2020 Aug 6.

14. Lu Y, Sun K, Guo S, Wang J, Li A, Rong X, Wang T, Shang Y, Chang W, Wang S. Early warning indicators of severe COVID-19: A single-center study of cases from Shanghai, China. Frontiers in medicine. 2020 Jul 17;7:432.

15. Malik P, Patel U, Mehta D, Patel N, Kelkar R, Akrmah M, Gabrilove JL, Sacks H. Biomarkers and outcomes of COVID-19 hospitalisations: systematic review and meta-analysis. BMJ evidence-based medicine. 2021 Jun 1;26(3):107-8.

16. Leulseged TW, Hassen IS, Ayele BT, Tsegay YG, Abebe DS, Edo MG, Maru EH, Zewde WC, Naylor LK, Semane DF, Dresse MT. Laboratory biomarkers of COVID-19 disease severity and outcome: Findings from a developing country. Plos one. 2021 Mar 15;16(3):e0246087.

17. Topp G, Bouyea M, Cochran-Caggiano N, Ata A, Torres P, Jacob J, Wales D. Biomarkers Predictive of Extubation and Survival of COVID-19 Patients. Cureus. 2021 Jun 5;13(6).

18. World Health Organization. WHO delivers advice and support for older people during COVID-19. World Health Organization. 2020 Aug. [Cited 30 September 2021]. Available from: https://www.who.int/news-room/feature-stories/detail/who-delivers-advice-and-support-for-older-people-during-covid-19

19. Center for Disease Control and Prevention. [Cited 2 October 2021]. Available from: https://www.cdc.gov/aging/covid19/covid19-older-adults.html

20. Ferguson J, Rosser JI, Quintero O, Scott J, Subramanian A, Gumma M, Rogers A, Kappagoda S. Characteristics and outcomes of coronavirus disease patients under nonsurge conditions, Northern California, USA, March–April 2020. Emerging infectious diseases. 2020 Aug;26(8):1679.

21. Pant S, Subedi M. Impact of COVID-19 on the elderly. Journal of Patan Academy of Health Sciences. 2020 Sep 15;7(2):32-8.

22. Guan WJ, Ni ZY, Hu Y, Liang WH, Ou CQ, He JX, Liu L, Shan H, Lei CL, Hui DS, Du B. Clinical characteristics of coronavirus disease 2019 in China. New England journal of medicine. 2020 Apr 30;382(18):1708-20.

23. Huang C, Wang Y, Li X, Ren L, Zhao J, Hu Y, Zhang L, Fan G, Xu J, Gu X, Cheng Z. Clinical features of patients infected with 2019 novel coronavirus in Wuhan, China. The lancet. 2020 Feb 15;395(10223):497-506.

24. Zheng F, Tang W, Li H, Huang YX, Xie YL, Zhou ZG. Clinical characteristics of 161 cases of corona virus disease 2019 (COVID-19) in Changsha. Eur Rev Med Pharmacol Sci. 2020 Mar 1;24(6):3404-10.

25. Zhao XY, Xu XX, Yin HS, Hu QM, Xiong T, Tang YY, Yang AY, Yu BP, Huang ZP. Clinical characteristics of patients with 2019 coronavirus disease in a non-Wuhan area

of Hubei Province, China: a retrospective study. BMC infectious diseases. 2020 Dec;20(1):1-8.

26. Wan S, Xiang YI, Fang W, Zheng Y, Li B, Hu Y, Lang C, Huang D, Sun Q, Xiong Y, Huang X. Clinical features and treatment of COVID-19 patients in northeast Chongqing. Journal of medical virology. 2020 Jul;92(7):797-806.

27. Khamis F, Al-Zakwani I, Al Naamani H, Al Lawati S, Pandak N, Omar MB, Al Bahrani M, Bulushi ZA, Al Khalili H, Al Salmi I, Al Ismaili R. Clinical characteristics and outcomes of the first 63 adult patients hospitalized with COVID-19: an experience from Oman. Journal of infection and public health. 2020 Jul 1;13(7):906-13.

28. Deng M, Qi Y, Deng L, Wang H, Xu Y, Li Z, Meng Z, Tang J, Dai Z. Obesity as a potential predictor of disease severity in young COVID-19 patients: a retrospective study. Obesity. 2020 Oct;28(10):1815-25.

29. Mikami T, Miyashita H, Yamada T, Harrington M, Steinberg D, Dunn A, Siau E. Risk factors for mortality in patients with COVID-19 in New York City. Journal of general internal medicine. 2021 Jan;36(1):17-26.

30. Shahriarirad R, Khodamoradi Z, Erfani A, Hosseinpour H, Ranjbar K, Emami Y, Mirahmadizadeh A, Lotfi M, Yeganeh BS, Nejad AD, Hemmati A. Epidemiological and clinical features of 2019 novel coronavirus diseases (COVID-19) in the South of Iran. BMC infectious diseases. 2020 Dec;20(1):1-2.

31. Li X, Xu S, Yu M, Wang K, Tao Y, Zhou Y, Shi J, Zhou M, Wu B, Yang Z, Zhang C. Risk factors for severity and mortality in adult COVID-19 inpatients in Wuhan. Journal of Allergy and Clinical Immunology. 2020 Jul 1;146(1):110-8.

32. Zhou F, Yu T, Du R, Fan G, Liu Y, Liu Z, Xiang J, Wang Y, Song B, Gu X, Guan L. Clinical course and risk factors for mortality of adult inpatients with COVID-19 in

Wuhan, China: a retrospective cohort study. The lancet. 2020 Mar 28;395(10229):1054-62.

33. Colaneri M, Sacchi P, Zuccaro V, Biscarini S, Sachs M, Roda S, Pieri TC, Valsecchi P, Piralla A, Seminari E, Di Matteo A. Clinical characteristics of coronavirus disease (COVID-19) early findings from a teaching hospital in Pavia, North Italy, 21 to 28 February 2020. Eurosurveillance. 2020 Apr 23;25(16):2000460.

34. Goyal P, Choi JJ, Pinheiro LC, Schenck EJ, Chen R, Jabri A, Satlin MJ, Campion Jr TR, Nahid M, Ringel JB, Hoffman KL. Clinical characteristics of Covid-19 in New York city. New England Journal of Medicine. 2020 Jun 11;382(24):2372-4.

35. Peckham H, de Gruijter NM, Raine C, Radziszewska A, Ciurtin C, Wedderburn LR, Rosser EC, Webb K, Deakin CT. Male sex identified by global COVID-19 meta-analysis as a risk factor for death and ITU admission. Nature communications. 2020 Dec 9;11(1):1-0.

36. Fan Z, Chen L, Li J, Cheng X, Yang J, Tian C, Zhang Y, Huang S, Liu Z, Cheng J. Clinical features of COVID-19-related liver functional abnormality. Clinical Gastroenterology and Hepatology. 2020 Jun 1;18(7):1561-6.

37. Cai Q, Huang D, Yu H, Zhu Z, Xia Z, Su Y, Li Z, Zhou G, Gou J, Qu J, Sun Y. COVID-19: abnormal liver function tests. Journal of hepatology. 2020 Sep 1;73(3):566-74.

38. Lei F, Liu YM, Zhou F, Qin JJ, Zhang P, Zhu L, Zhang XJ, Cai J, Lin L, Ouyang S, Wang X. Longitudinal association between markers of liver injury and mortality in COVID-19 in China. Hepatology. 2020 Aug;72(2):389-98.

39. Chen T, Wu DI, Chen H, Yan W, Yang D, Chen G, Ma K, Xu D, Yu H, Wang H, Wang T. Clinical characteristics of 113 deceased patients with coronavirus disease 2019: retrospective study. bmj. 2020 Mar 26;368.

40. Liu W, Tao ZW, Wang L, Yuan ML, Liu K, Zhou L, Wei S, Deng Y, Liu J, Liu HG, Yang M. Analysis of factors associated with disease outcomes in hospitalized patients with 2019 novel coronavirus disease. Chinese medical journal. 2020 May 5;133(9):1032.

41. Aziz M, Fatima R, Lee-Smith W, Assaly R. The association of low serum albumin level with severe COVID-19: a systematic review and meta-analysis. Critical Care. 2020 Dec;24:1-4.

42. Italian report on kidney disease. [Cited 2 October 2021]. Available from: https://www.epicentro.iss.it/coronavirus/bollettino/Report-COVID-201917marzo-2.pdf

43. Henry BM, Aggarwal G, Wong J, Benoit S, Vikse J, Plebani M, Lippi G. Lactate dehydrogenase levels predict coronavirus disease 2019 (COVID-19) severity and mortality: a pooled analysis. The American journal of emergency medicine. 2020 Sep 1;38(9):1722-6.

44. Yan L, Zhang HT, Goncalves J, Xiao Y, Wang M, Guo Y, Sun C, Tang X, Jing L, Zhang M, Huang X. An interpretable mortality prediction model for COVID-19 patients. Nature machine intelligence. 2020 May;2(5):283-8.

45. Han Y, Zhang H, Mu S, Wei W, Jin C, Tong C, Song Z, Zha Y, Xue Y, Gu G. Lactate dehydrogenase, an independent risk factor of severe COVID-19 patients: a retrospective and observational study. Aging (Albany NY). 2020 Jun 30;12(12):11245.

46. Qin C, Ziwei MP, Tao SY, Ke PC, Shang MM. Dysregulation of immune response in patients with COVID-19 in Wuhan, China; Clinical Infectious Diseases; Oxford Academic. Clinical Infectious Diseases.

47. Henry BM, De Oliveira MH, Benoit S, Plebani M, Lippi G. Hematologic, biochemical and immune biomarker abnormalities associated with severe illness and mortality in coronavirus disease 2019 (COVID-19): a meta-analysis. Clinical Chemistry and Laboratory Medicine (CCLM). 2020 Jul 1;58(7):1021-8.

48. Ganji A, Farahani I, Khansarinejad B, Ghazavi A, Mosayebi G. Increased expression of CD8 marker on T-cells in COVID-19 patients. Blood Cells, Molecules, and Diseases. 2020 Jul 1;83:102437.

49. Lippi G, Plebani M, Henry BM. Thrombocytopenia is associated with severe coronavirus disease 2019 (COVID-19) infections: a meta-analysis. Clinica chimica acta. 2020 Jul 1;506:145-8.

50. Yin S, Huang M, Li D, Tang N. Difference of coagulation features between severe pneumonia induced by SARS-CoV2 and non-SARS-CoV2. Journal of thrombosis and thrombolysis. 2021 May;51(4):1107-10.

51. Qu R, Ling Y, Zhang YH, Wei LY, Chen X, Li XM, Liu XY, Liu HM, Guo Z, Ren H, Wang Q. Platelet-to-lymphocyte ratio is associated with prognosis in patients with coronavirus disease-19. Journal of medical virology. 2020 Sep;92(9):1533-41.

52. Du RH, Liang LR, Yang CQ, Wang W, Cao TZ, Li M, Guo GY, Du J, Zheng CL, Zhu Q, Hu M. Predictors of mortality for patients with COVID-19 pneumonia caused by SARS-CoV-2: a prospective cohort study. European Respiratory Journal. 2020 May 1;55(5).

53. Lippi G, Lavie CJ, Sanchis-Gomar F. Cardiac troponin I in patients with coronavirus disease 2019 (COVID-19): evidence from a meta-analysis. Progress in cardiovascular diseases. 2020 May;63(3):390.

54. Chan KH, Farouji I, Hanoud AA, Slim J. Weakness and elevated creatinine kinase as the initial presentation of coronavirus disease 2019 (COVID-19). The American journal of emergency medicine. 2020 Jul 1;38(7):1548-e1.

55. Zhang Q, Shan KS, Minalyan A, O'Sullivan C, Nace T. A rare presentation of coronavirus disease 2019 (COVID-19) induced viral myositis with subsequent rhabdomyolysis. Cureus. 2020 May;12(5).

56. Liu F, Li L, Xu M, Wu J, Luo D, Zhu Y, Li B, Song X, Zhou X. Prognostic value of interleukin-6, C-reactive protein, and procalcitonin in patients with COVID-19. Journal of clinical virology. 2020 Jun 1;127:104370.

57. Tan C, Huang Y, Shi F, Tan K, Ma Q, Chen Y, Jiang X, Li X. C-reactive protein correlates with computed tomographic findings and predicts severe COVID-19 early. Journal of medical virology. 2020 Jul;92(7):856-62.

58. Zhang J, Zhou L, Yang Y, Peng W, Wang W, Chen X. Therapeutic and triage strategies for 2019 novel coronavirus disease in fever clinics. The Lancet Respiratory Medicine. 2020 Mar 1;8(3):e11-2.

59. Chalmers S, Khawaja A, Wieruszewski PM, Gajic O, Odeyemi Y. Diagnosis and treatment of acute pulmonary inflammation in critically ill patients: the role of inflammatory biomarkers. World journal of critical care medicine. 2019 Sep 11;8(5):59.

60. Zhang L, Yan X, Fan Q, Liu H, Liu X, Liu Z, Zhang Z. D-dimer levels on admission to predict in-hospital mortality in patients with Covid-19. Journal of thrombosis and haemostasis. 2020 Jun;18(6):1324-9.

61. Lippi G, Favaloro EJ. D-dimer is associated with severity of coronavirus disease 2019: a pooled analysis. Thrombosis and haemostasis. 2020 May;120(05):876-8.

62. Yu B, Li X, Chen J, Ouyang M, Zhang H, Zhao X, Tang L, Luo Q, Xu M, Yang L, Huang G. Evaluation of variation in D-dimer levels among COVID-19 and bacterial pneumonia: a retrospective analysis. Journal of thrombosis and thrombolysis. 2020 Oct;50(3):548-57.

63. Tang N, Bai H, Chen X, Gong J, Li D, Sun Z. Anticoagulant treatment is associated with decreased mortality in severe coronavirus disease 2019 patients with coagulopathy. Journal of thrombosis and haemostasis. 2020 May;18(5):1094-9.

64. Wang D, Hu B, Hu C, Zhu F, Liu X, Zhang J, Wang B, Xiang H, Cheng Z, Xiong Y, Zhao Y. Clinical characteristics of 138 hospitalized patients with 2019 novel coronavirus–infected pneumonia in Wuhan, China. Jama. 2020 Mar 17;323(11):1061-9.

65. Zou Y, Guo H, Zhang Y, Zhang Z, Liu Y, Wang J, Lu H, Qian Z. Analysis of coagulation parameters in patients with COVID-19 in Shanghai, China. Bioscience trends. 2020.
