# Supplementary material for: Analysis of altered level of blood-based biomarkers in prognosis of COVID-19 patients
Source: PLoS One. 2023 Aug 4;18(8):e0287117. doi: 10.1371/journal.pone.0287117 (PMC10403103; doi:10.1371/journal.pone.0287117)
Supplement: Attachment — Submitted filename: Response to Reviewers.docx [file pone.0287117.s002.docx]

**Dear editor,**

Thank you for giving us the opportunity to submit a revised draft of our manuscript titled “Analysis of altered level of blood-based biomarkers in prognosis of COVID-19 patients” to PLOS ONE. We appreciate the time and effort that you and the four reviewers have dedicated to providing valuable feedback on our manuscript. We are grateful to the reviewers for their insightful comments on this study. We have been able to incorporate changes to reflect most of the suggestions provided by the reviewers. Please find enclosed our response to the reviewer’s comments.

| **Date:** | Mar 30 2022 11:54AM |
| --- | --- |
| **To:** | "Ajaya Basnet" xlcprk@gmail.com |
| **From:** | "PLOS ONE" plosone@plos.org |
| **Subject:** | PLOS ONE Decision: Revision required [PONE-D-22-06698] |
| **Attachment(s):** | [PONE-D-22-06698_reviewer.pdf](https://www.editorialmanager.com/pone/download.aspx?guid=%7b557f15c7-94cf-4e46-bc6d-ffb6c20f558c%7d&scheme=13&id=463327) |

PONE-D-22-06698

Analysis of altered level of blood-based biomarkers in the prognosis of COVID-19 patients

Dear Dr. Basnet,

Thank you for submitting your manuscript to PLOS ONE. After careful consideration, we feel that it has merit but does not fully meet PLOS ONE’s publication criteria as it currently stands. Therefore, we invite you to submit a revised version of the manuscript that addresses the points raised during the review process.

**General comments**

1. Please ensure that your manuscript meets PLOS ONE's style requirements, including those for file naming. The PLOS ONE style templates can be found at

https://journals.plos.org/plosone/s/file?id=wjVg/PLOSOne_formatting_sample_main_body.pdf and

https://journals.plos.org/plosone/s/file?id=ba62/PLOSOne_formatting_sample_title_authors_affiliations.pdf

**Response:** We thank you for this useful suggestion. We followed the links and made the necessary changes to the manuscript. As suggested, upon improvements, we have prepared and named three different files as 'Response to Reviewers' (for rebuttal letter), and ' Manuscript with Track Changes’ (for a marked-up copy of the manuscript), and ‘Revised Manuscript’ (for revised paper). Additionally, we have checked and attest that all formatting and style requirements have been met in the manuscript.

2. If you would like to make changes to your financial disclosure, please include your updated statement in your cover letter. Guidelines for resubmitting your figure files are available below the reviewer comments at the end of this letter.

If applicable, we recommend that you deposit your laboratory protocols in protocols.io to enhance the reproducibility of your results. Protocols.io assigns your protocol its own identifier (DOI) so that it can be cited independently in the future. For instructions see: <https://journals.plos.org/plosone/s/submission-guidelines#loc-laboratory-protocols>. Additionally, PLOS ONE offers an option for publishing peer-reviewed Lab Protocol articles, which describe protocols hosted on protocols.io. Read more information on sharing protocols at <https://plos.org/protocols?utm_medium=editorial-email&utm_source=authorletters&utm_campaign=protocols>.

**Response:** The financial disclosure for the manuscript remains the same. Hence, the cover letter has not been updated. We thank you for providing us with the direct link to resubmit the figure files of the manuscript. Deposition of the study laboratory protocols is inapplicable for this study.

3. You indicated that you had ethical approval for your study. In your Methods section, please ensure you have also stated whether you obtained consent from parents or guardians of the minors included in the study or whether the research ethics committee or IRB specifically waived the need

**Response:** We added a sentence in the “Data collection” section of Methodology to indicate that we had ethical approval for the study and that the need for patient and/or relative consent was waived.

4. We note that you have indicated that data from this study are available upon request. PLOS only allows data to be available upon request if there are legal or ethical restrictions on sharing data publicly. For more information on unacceptable data access restrictions, please see

http://journals.plos.org/plosone/s/data-availability#loc-unacceptable-data-access-restrictions.

In your revised cover letter, please address the following prompts:

a) If there are ethical or legal restrictions on sharing a de-identified data set, please explain them in detail (e.g., data contain potentially sensitive information, data are owned by a third-party organization, etc.) and who has imposed them (e.g., an ethics committee). Please also provide contact information for a data access committee, ethics committee, or other institutional body to which data requests may be sent.
b) If there are no restrictions, please upload the minimal anonymized data set necessary to replicate your study findings as either Supporting Information files or to a stable, public repository and provide us with the relevant URLs, DOIs, or accession numbers. For a list of acceptable repositories, please see http://journals.plos.org/plosone/s/data-availability#loc-recommended-repositories.

We will update your Data Availability statement on your behalf to reflect the information you provide.

**Response:** We thank you for this useful suggestion. Since there are no restrictions in sharing the anonymized data set of the study, we have uploaded the dataset in Dryad. For accessing the dataset, kindly visit the links

<https://datadryad.org/stash/share/RIQyCjUIzcc35aAmTV6p7UuFzj23Xk66tW6BmZUBaaw>

Or

<https://doi.org/10.5061/dryad.cjsxksn7r>

As mentioned in comment point (a), there are not any ethical or legal restrictions on sharing a de-identified data set; hence, there are no changes in the cover letter too.

**Response to Reviewer 1**

1. Analysis of altered level of blood-based biomarkers in the prognosis of COVID-19 patient’s manuscript is written well in criteria adherence with PLOS ONE policy. Differentiation between two groups needs to be addressed more.

**Response:** First of all, we will like to express our sincere gratitude for your kind suggestions. We appreciate the time and effort that you dedicated to providing feedback on our manuscript and are grateful for the insightful comments on our paper.

Thank you for pointing this out. We agree with this comment. Therefore, we have categorized the study population into two groups, including patients with COVID-19 and patients without COVID-19. Patients with COVID-19 were the individuals diagnosed with COVID-19 by real-time reverse transcriptase-polymerase chain reaction (rtRT-PCR) in the nasopharyngeal swab samples. Patients without COVID-19 were the individuals who had visited the hospital for a general check-up and had not any symptoms similar to individuals with COVID-19. Additionally, when they were tested for COVID-19 by rtRT-PCR in the nasopharyngeal swab samples, they were not diagnosed with COVID-19, and hence were considered as “control” in the study. Kindly refer to the “study population and design” section of the methodology (lines number 114-120).

**Response to Reviewer 2**

The premise of the study is good and has potential clinical value, but the data and analysis presented contain several errors and incomplete information. Please address the following issues:

1. Please define the cases and controls clearly in the study design. There is no mention of controls in the study design at all. Are they healthy controls? Are they people who tested negative for COVID-19? Are they patients admitted to the hospital, and if so, for what reason? Why did you decide to use this group of individuals as controls?

**Response:** First of all, we will like to express our sincere gratitude for your kind suggestions. We appreciate the time and effort that you dedicated to providing feedback on our manuscript and are grateful for the insightful comments on our paper.

Agreeing with the comment, we have now updated our manuscript with the distinct details of both the study population (patient with COVID-19) and control (patient without COVID-19). Kindly refer to the “study population and design” section of the methodology (lines number 114-120).

2. If available, please include the severity of COVID-19 in the patients, whether it was moderate or severe, and whether they needed ICU admission or mechanical ventilation, as this has an impact on the prognosis.

**Response:** Thank you for this suggestion. Our main attempt in this manuscript was to identify the most significant blood-based laboratory biomarkers among the patients with COVID-19. Severity classification for the COVID-19, as per WHO guidelines relies upon the clinical parameters (SpO2, PiO2/FiO2 ratio, respiratory rate) and also the radiological findings of patchy shadowing, laterality, and types of abnormality (interstitial, ground-glass opacity, and others). We were not able to retrieve such clinical and radiological details in a needful manner from the respective departments. Either the required cases were missing or most of them were incomplete, which was also a similar case for patients' need for ICU admission or mechanical ventilation. Such poor data management could be due to the hassle and overexertion amongst the healthcare workers to diagnose and treat the patients with COVID-19. However, with the insights generated from this suggestion, we have added these contributing factors as a limitation of the study (line number 338-340).

3. Did you include all patients who met the inclusion criteria in the study, and if not, how was the sampling done? Did you estimate a sample size beforehand that would give adequate power to the study? How did you decide on 500 controls?

**Response:** You have raised an important point here. Yes, we included all patients who met the inclusion criteria in the study. Patients infected with SARS-CoV-2, diagnosed with COVID-19, and then hospitalized in NAPFH within the study period (April 2021 to September 2021) were included.

The sample size for the study was calculated as per the prevalence of COVID-19 in Nepal reported by the Ministry of Health and Population of Nepal. Initially, the sample size (patients with COVID-19) was 225 for the study. However, with the increasing case of patients with SARS-CoV-2 infection, the hospital had abundant patients diagnosed with COVID-19. Therefore, in order to make the data more generalizable among the Nepalese population, we included all COVID-19-diagnosed patients admitted to the hospital between April 2021 to September 2021. Similarly, initially, the study also planned to compare the findings with 225 control. Later, while the number of patients with COVID-19 increased substantially, the patients without COVID-19 also increased (unsubstantially as compared to patients with COVID-19). However, we barely managed to have the control group involving 500 participants within the study period. This might be due to the majority of the population residing at home to protect themselves from acquiring SARS-CoV-2 infection. The latter changes in the sample size of the study were also authorized by the institutional review committee of the Shi-Gan Health Foundation.

4. Please mention when the lab measurements were taken, was it upon admission or during the admission period? Was it taken at the same phase of the admission period for all patients? How did you deal with cases in which lab measurements were repeated during the admission period?

**Response:** Once the suspected patients visited the hospital, they were sampled (nasopharyngeal swab) for diagnosis of COVID-19 by rtRT-PCR. Patients in need of hospitalization were judged by clinicians and were admitted accordingly. The report for rtRT-PCR test was made available only after 24 hours. The laboratory measurements for all 1,232 patients with COVID-19 (1,232 samples) were taken upon admission, within 24 hours of hospitalization, who were later (after 24 hours) confirmed, if they were diagnosed with COVID-19. The rest of the laboratory measurements (n=548) from some of the same patients were taken, within 36 hours of hospitalization. Such immediate sampling was performed once the patients were affirmed for SARS-CoV-2 infection to monitor the patient's condition. Such random sampling was also performed as per the clinicians' judgment, which might be based either on worsening of patients' health condition or for any other clinical reasons. Such immediate sampling would not have altered the blood-based biomarkers substantially; since the exact treatment regimen for the treatment of COVID-19 patients was not initiated.

Because this study focuses on identifying the significant laboratory biomarkers in blood samples of patients with COVID-19, we included the repeated samples in the study. Such samples were also of significant value in identifying the predictors of disease, as they belonged to the target study population at the initial phase.

5. Several of the lab values vary hugely with age, especially in the pediatric population. What is your rationale for including patients of all ages in the same analysis, instead of using more strict inclusion criteria or analyzing the pediatric population separately?

**Response:** The American Academy of Pediatrics has identified the upper age limit for the pediatric population as 21 years. In this study, the pediatric population makes up a lesser proportion of total COVID-19 patients as compared to others (3.73% versus 96.27%). Hence, including patients of all ages in the same analysis would not have significantly affected the values.

6. Please create a separate heading under the results for mortality.

When you say "The COVID-19 patients (n = 58) with elevated levels of glucose (p=0.007)....", please specify what you are comparing them to when you give p values for deceased patients? Is it with other COVID patients who survived, or is it with controls?

**Response:** A separate table including the analysis for significant biomarkers among the COVID-19 patients, who either recovered or succumbed to the disease, was added to the result section.

Thank you for these valuable suggestions. We have now added a table (Table 3) (line no 212) along with the description (line no. 203-211) of the table, preceding the table, in the result section of the manuscript. Currently, the table coherently indicates the comparison in the mentioned sentence was between the deceased and recovered patients with COVID-19.

7. Please mention the period of follow-up, since this is necessary to interpret mortality data. Is it in-hospital mortality, or mortality at a certain point in the follow-up period (e.g. 30-day mortality)? Please elaborate on the causes of death if possible.

**Response:** All reported mortalities of COVID-19 patients were in-hospital mortality. We defined in-hospital mortality as the percentage of patients with COVID-19 who died in the hospital during the treatment course.

Thank you for your suggestion concerning the elaboration of the cause of death. It would have been interesting to explore this aspect. However, in case of this study, it seems slightly out of scope because we are only identifying the significant laboratory biomarkers for the prognosis of COVID-19 patients and their variations among the study groups. Nonetheless, as per the data of this study, the majority of patients with COVID-19 died because of multiple organ dysfunction syndromes (n=23, 39.66%), followed by severe sepsis-related MODS (n=17, 29.31%), refractory hypoxemia with septic shock (n=9, 15.52%), type 2 respiratory failure with septicemia (n=6, 10.34%), and septic shock with acute myocarditis (n=3, 5.17%). Additionally, the correlation of laboratory biomarkers with such causes of death may be out of scope considering the study title. However, further, we would also like to request the reviewer to provide his/her thoughts one more time about the incorporation of causes of death in the manuscript by considering the findings.

8. In the second paragraph of the discussion section, "This study also found that COVID-19 patients ...... were at a higher risk of COVID-19 infection...." Please rephrase this.

**Response:** Thank you for your suggestion. We have revised and rephrased the mentioned sentence (Line number 221-222).

9. As it stands, the findings do not justify your conclusion, especially regarding the prognosis. Please rewrite the conclusion to better reflect your findings, or add to your findings to better support your conclusion.

**Response:** The conclusion has been readdressed based upon the modifications after the reviewers’ suggestions and as per the study findings (line no 358-365).

10. Please make all data available for review as per the journal policy.

**Response:** Data of the study are made available as per the journal policy. Links for the dataset are as follows

<https://datadryad.org/stash/share/RIQyCjUIzcc35aAmTV6p7UuFzj23Xk66tW6BmZUBaaw>

Or

<https://doi.org/10.5061/dryad.cjsxksn7r>

**Response to Reviewer 3**

1. Thanks to the authors. There are some areas that need to be corrected in this study. Minor revision required. The introduction part of the article is too long and should be shortened. The discussion part of the article is too long and should be shortened.

**Response:** Thank you for your kind suggestions. We have made several changes in the manuscript based on the suggestions from the editor and reviewers.

Based on your suggestions, we have reduced the length of both the introduction (shortened by 164 words) and discussion (shortened by 308 words) parts. However, we would also sincerely request you to acknowledge that the discussion part of the manuscript needs to thoroughly address approximately 30 study variables, along with the support of reference articles to provide better insights to the readers. Hence, the discussion section is lengthier as compared to rest of the sections. We have also reduced the word counts in the manuscript, apart from introduction and discussion.

**Response to Reviewer 4**

1. Dear Authors, this research, which investigated biomarkers in the Covid-19 pandemic, does not make a current contribution to the literature. I think that the article is not of scientific quality at the level of publication.

**Response:** Thank you for giving your precious time and suggestions to our manuscript.

We agree with the reviewer about the study's potential to make a significant contribution to the literature in the current period. However, we would like to highlight some of the facts.

Though the decline in case numbers of patients acquiring SARS-CoV-2 infection has been observed throughout the world, the incidences of patients acquiring SARS-CoV-2 infection are not nil. With the incessant emergence of SARS-CoV-2 variants such as Stealth, Deltacron, NeoCov, and others, WHO reports more than 0.4 million new cases COVID-19 patients with thousands of death in a global basis, daily. Similarly, in context of Nepal, the Ministry of Health and Population of Nepal reports new cases of patients with COVID-19 every day along with the incidences of in-hospital.

To the best of our knowledge, this is the first study from Nepal to unfold the significance of specific blood-based biomarkers along with their varying serum levels when compared to the control group. We also request the reviewer to acknowledge the fact that articles analyzing and summarizing the significance of all biochemical, hematological, coagulatory, inflammatory, and cardiac biomarkers are still limited in the global scientific community. This was the major reason among the authors to pursue this study too. With the handful of such published literature, we believe this study will contribute to the scientific community by forming a basis to write the review articles by the researchers or scientists residing in different parts of the world.

With the limited technology available during the 1918 Influenza pandemic, there was very limited literature available concerning the Influenza, be it secondary bacterial infection among the infected patients, laboratory biomarkers for a better prognosis, or significant cause of death. We believe this manuscript will make a significant contribution to such preexisting literature and in the future may serve as a valuable piece of intellect to the researchers of upcoming generations.

Lastly, we have improved the article based on the suggestions from the editor and the rest of the reviewers. Addressing the comments has improved the quality of the manuscript. Besides all the research authors have gone through the manuscript to improve the quality of the article, including, grammar, data analysis and presentation, and others. Therefore, we sincerely request the reviewer to review the improved article and let us provide valuable suggestions.
